# Ruxolitinib treatment permits lower cumulative glucocorticoid dosing in children with secondary hemophagocytic lymphohistiocytosis

**DOI:** 10.1186/s12969-021-00534-0

**Published:** 2021-04-01

**Authors:** Ying Chi, Rong Liu, Zhi-xuan Zhou, Xiao-dong Shi, Yu-chuan Ding, Jian-guo Li

**Affiliations:** Children’s Hospital Affiliated to the Capital Institute of Pediatrics, Beijing, 100020 China

**Keywords:** Ruxolitinib, Hemophagocytic lymphohistiocytosis, Janus kinase inhibitor, Children

## Abstract

**Background:**

This study aimed to analyze the effects of ruxolitinib on children with secondary hemophagocytic lymphohistiocytosis (HLH).

**Methods:**

Eleven pediatric patients diagnosed with HLH and treated with ruxolitinib (ruxolitinib group: group R) between November 2017 and August 2018 were retrospectively analyzed. Eleven age-matched pediatric patients with HLH undergoing conventional treatment (control group: group C) during the same period were also analyzed.

**Results:**

In group R, three patients who did not respond to methylprednisolone (MP) pulse and intravenous immunoglobulin (IVIG) therapies were treated with Ruxolitinib and their temperature decreased to normal levels. Four patients had normal temperature after conventional treatment (dexamethasone and etoposide, with or without cyclosporine A), but they had severe organ involvement, including obvious yellowing of the skin, increased liver enzyme levels and neuropsychiatric symptoms, and they were all ameliorated with ruxolitinib treatment. Four patients were relieved with ruxolitinib therapy alone. In group C, the body temperatures of eleven patients decreased to normal levels after conventional treatment. The body temperature of group R patients decreased to normal levels more rapidly than that of group C patients. The glucocorticoid dosage in group R was significantly lower than that in group C. Both groups were followed-up for 2–2.5 years. No obvious adverse drug reactions to ruxolitinib were observed during treatment and follow-up.

**Conclusion:**

Ruxolitinib might be an effective drug in controlling body temperature and reducing inflammation indicators. It might be a potential replacement for glucocorticoid therapy for HLH treatment in children, thereby reducing or avoiding glucocorticoid-related adverse reactions.

## Background

Hemophagocytic syndrome is also known as hemophagocytic lymphohistiocytosis (HLH). This rare life-threatening syndrome is characterized by excessive proliferation and activation of lymphocytes caused by cytokine storms and severe systemic inflammatory responses. According to the accepted doctrine, the pathogenesis of HLH is closely associated with the “cytokine storm” [1]. Depending on the etiology, there are two forms of HLH: primary autosomal recessive inheritance, also known as familial hemophagocytic lymphohistiocytosis, and secondary HLH, which develops because of strong immune activation. Primary HLH is mostly caused by genetic defects leading to immune system dysfunction. Infection, connective tissue disease, and malignancy are considered common causes of secondary HLH [2–4]. Epstein-Barr virus (EBV), a DNA virus and member of the *Herpesviridae* family, has been consistently associated with HLH [5–15].

HLH in children is a rare disease with a high fatality rate. Studies have shown that most cytokines related to HLH are activated through activation of the Janus Kinase (JAK)/signal transducer and activator of transcription signaling pathway, which not only regulates the biological activity of cytokines, but also affects the differentiation of primary T cells into T helper (TH) cell families, TH1, TH2, TH17, and regulatory T cells [16].

The above clinical and laboratory findings are related to the pathophysiology of HLH. High interleukin levels cause fever. Elevated ferritin > 10,000 μg/L has been demonstrated to be 90% sensitive and 96% specific for HLH [17–20]. Activation of lymphocytes can result in high concentrations of soluble IL-2 receptor [21].

Most clinicians still adopt the HLH-04 protocol recommended by the Histiocyte Society for the treatment of HLH [22]. Conventional treatment options typically include three phases of induction therapy, maintenance therapy, and/or hematopoietic stem cell transplantation. The drugs for HLH include dexamethasone, cyclosporine A, and etoposide. However, a multi-center study in 2016 revealed no significant benefit from cyclosporine and intrathecal injections, and macrophage activation syndrome secondary to connective tissue disease is not always treated with the HLH-04 regimen. The five-year survival rate for secondary HLH in adults worldwide is approximately 54% and the survival rate reported in China is even lower, ranging from 31.7–56.1% [23, 24]. Hence, less toxic, more effective, and better targeted immunosuppressive treatments in HLH are urgently needed.

In recent years, JAK inhibitors have been the focus of research on new small molecule targeted therapies and can be used for the treatment of inflammatory diseases such as hematological diseases, tumors, rheumatoid arthritis, ulcerative colitis, and other autoimmune diseases [25]. Ruxolitinib is a Janus-associated kinase 1/2 (JAK1/2) inhibitor that impedes downstream signaling pathways of cytokines such as interferon-γ, IL-2, and IL-6 to reduce inflammatory responses triggered by these cytokines, which play important roles in HLH.

Treatment of children with secondary HLH is an off-label use for ruxolitinib because it is only used for myelofibrosis [26], polycythemia [27], and graft-versus-host disease (GVHD) [28–30]. But ruxolitinib was found to control inflammatory storms and prolong survival in secondary HLH model mice; this treatment was also effective in 10 cases of HLH [31–35], including one child from the USA [33].

Herein, we present 11 cases of children treated with ruxolitinib. We compared the effects of ruxolitinib administration in children with HLH to the effects of conventional therapy in a control group of children with HLH; both groups were followed up for 2–2.5 years.

## Materials and methods

### Patients

A study was performed on 11 children diagnosed with HLH and treated with ruxolitinib (group R) and 11 children with HLH who were age-matched and were not administered ruxolitinib during the same period (group C). The diagnosis was made between November 2017 and August 2018 with a follow-up endpoint in February 2020. This study was reviewed by the Hospital Ethics Committee; informed consent was obtained from the patients’ parents, who had signed a written instrument, prior to the use of ruxolitinib and specimen collection.

### Inclusion criteria

Compliance with HLH-2004 diagnostic criteria [2] was the inclusion criterion for this study. A diagnosis of HLH can be made if five of the following eight criteria are fulfilled: (1) fever; (2) splenomegaly; (3) cytopenias (affecting ≥2 of three lineages in peripheral blood, hemoglobin < 90 g/L, platelets < 100 × 10^9^/L, neutrophils < 1.0 × 10^9^/L); (4) hypertriglyceridemia and/or hypofibrinogenemia; (5) hemophagocytosis in the bone marrow, spleen or lymph nodes (no evidence of malignancy); (6) low or absent natural killer (NK) cell activity (according to local laboratory reference); (7) ferritin ≥500 μg/L; and (8) soluble cluster of differentiation 25 (i.e., soluble interleukin-2 (IL-2) receptor (IL-2R)) ≥ 2400 U/ml.

For HLH in combination with rheumatological disease, such as juvenile idiopathic arthritis (systemic type), is classified as macrophage activation syndrome (MAS) if the following criteria are met [36, 37]: Serum ferritin > 684 ng/ml, as well as any two of the following: (1) Platelet count ≤181 × 10^9^/L; (2) aspartate aminotransferase > 48 U/L; (3) triglycerides > 156 mg/dL; (4) fibrinogen ≤ c360 mg/dL.

### Exclusion criteria

Children underwent purified protein derivative skin test, chest radiography, high-resolution computed tomography if necessary, and T-SPOT (T-cell enzyme immuno-spotting) to confirm the absence of tuberculosis infection, which was the exclusion criterion for this study.

### Etiological analysis

All children were tested for bacteria, viruses, fungi, and parasites. Whole exome sequencing was performed for all cases in group R and no known pathogenic genes were found.

### Treatment

In group R, in seven patients ruxolitinib was added to therapies that had previously failed, and four patients were treated with ruxolitinib alone immediately after diagnosis of HLH. The dosage used in this study was based on the lower dose used for GVHD in children [38], which, similar to HLH, is characterized by the production of high levels of proinflammatory cytokines. The doses were 2.5 mg/dose orally twice daily for those with body weight ≤ 25 kg and 5 mg/dose orally twice daily for those with body weight > 25 kg [30]. The use of ruxolitinib was discontinued after 3 months of administration. Children with viral infections were also treated with antiviral therapy. Group C was treated with the conventional treatment (dexamethasone and etoposide, with or without cyclosporine A).

### Follow-up

Monitoring of clinical symptoms, such as body temperature, hepatosplenomegaly, dizziness, headache, rash, dyspnea, gastrointestinal reaction was performed. In addition, the following parameters were determined: routine blood test results (white blood cells, hemoglobin, platelets, etc.), coagulation function, C-reactive protein, ferritin, cytokines, infections (i.e., tuberculosis, adenovirus, Epstein-Barr virus, cytomegalovirus and fungal infections), renal function (serum creatinine, urea nitrogen), liver function (ALT, AST, GGT, ALB, TBIL, I-BIL, and D-BIL). The children were followed-up in outpatient clinics to record their clinical symptoms, treatment status, and outcomes once every month for the first, second, and third months, and then once every 3 months, and once every 6 months after 1 year, for a total of 2–2.5 years.

### Safety evaluation

Symptoms of discomfort after taking medication were recorded. Liver and kidney functions, as well as whether the patients had co-infections were monitored.

### Statistical analysis

SPSS Statistics 24 (SPSS, Inc., Chicago, IL, USA) was used for statistical analysis. For continuous data, normal distribution was expressed as the means ± standard deviations and the independent sample *t*-test was used. Data that did not meet the normal distribution were expressed as the median (P25, P75), and the Wilcoxon rank-sum test was used. For continuous data from ≥3 measurements, the repeated-measures design data analysis of variance was used. Categorical variables were represented by N (%), and chi-square test was used. *P* <  0.05 was considered statistically significant.

## Results

### General information

Group R consisted of 11 children: six girls (54.5%) and five boys (45.5%); aged 1–6 years, with a median age of 3.3 years. The course of HLH prior to ruxolitinib administration varied from 4 days to 2 months. Group C consisted of 11 children: five girls (45.5%) and 6 boys (54.5%); aged 1–8 years, with a median age of 3.8 years. There was no significant difference in the age and sex of the children in the two groups. Information on clinical features, underlying diseases, and therapy of the two groups is shown in Table 1.

**Table 1:** Clinical features, underlying diseases, and therapy of the two groups of patients

		Group R number [percentage (%)]	Group C number [percentage (%)]
Clinical features	Fever ≥38.5 °C	11 (100)	11 (100)
	Splenomegaly	11 (100)	11 (100)
	Cytopenias	11 (100)	10 (90.91)
	Hypofibrinogenemia/ Hypertriglyceridemia	11 (100)	10 (90.91)
	Elevated ferritin	11 (100)	11 (100)
	Hemophagocytosis	7 (63.64)	7 (63.64)
	Low or absent NK-cell activity	4 (36.36)	7 (63.64)
	Elevated soluble CD25	7 (63.64)	7 (63.64)
Underlying diseases	Infection	8 (72.73)	4 (36.36)
	Juvenile idiopathic arthritis (systemic type)	2 (18.18)	6 (54.55)
	Kawasaki disease	1 (9.10)	/
	Systemic lupus erythematosus	/	1 (9.10)
Therapy (Group R: before ruxolitinib)	HLH-04 MP and IVIG	4 (36.36) 3 (27.27)	6 (54.55) 5 (45.45)
	IVIG	4 (36.36)	/

*NK-cell* natural killer cell; *HLH* hemophagocytic lymphohistiocytosis; *MP* methylprednisolone; *IVIG* intravenous immunoglobulin; *Group R* ruxolitinib-treated group; *Group C* control group treated with conventional therapy

### Etiology

In group R, 8 cases (72.72%) were caused by infection, including 5 cases of Epstein-Barr virus (EBV) infection, 1 case of hepatitis B virus (HBV) infection (Li et al., in press), 1 case of cytomegalovirus infection, and 1 case of influenza virus infection; the other 3 cases had autoimmunity diseases, there were two cases (18.18%) of juvenile idiopathic arthritis (systemic type), and one case (9.1%) of Kawasaki disease. In group C, 4 cases (36.36%) were caused by infection, including 2 cases of EB virus infection and 2 cases of parainfluenza virus infection. Six cases (54.54%) with no infection manifested as juvenile idiopathic arthritis (systemic type), and 1 case (9.1%) with systemic lupus erythematosus. In all 11 cases of group R, no known gene mutation was detected by whole exon gene analysis, which was considered as secondary HLH.

### Severe organ involvement

In group R, R4 had obvious skin yellowing, liver damage, and cholestasis; R6 and R7 had CNS involvement such as convulsions, drowsiness, and coma; R6 also had pulmonary hemorrhage and gastrointestinal bleeding. In group C, C4 had liver damage, coronary artery dilation, and pulmonary hypertension; C9 had bronchopneumonia, thrush, and febrile convulsions.

### Efficacy of ruxolitinib therapy in group R

**Fig. 1:**
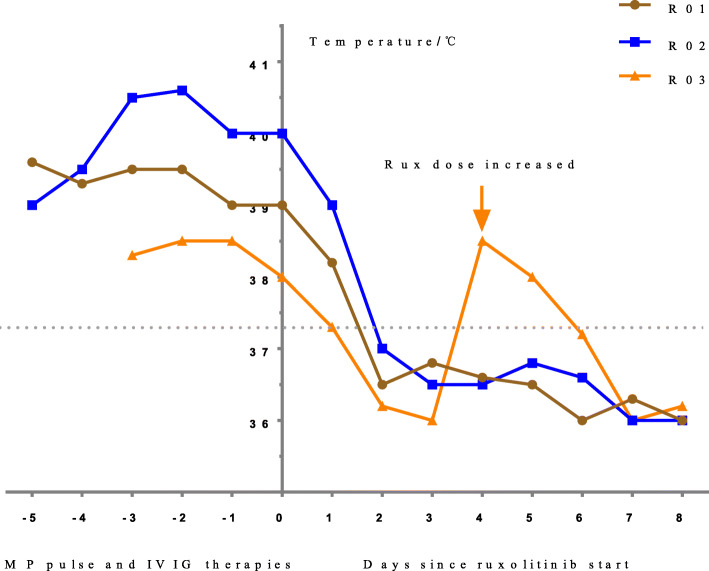
Daily temperature peak of patients (R1-R3) in the hospital. The body temperature rapidly returned to normal after 2 days of ruxolitinib treatment. Abbreviations: Rux, ruxolitinib; MP, methylprednisolone; IVIG, intravenous immunogloblin

**Fig. 2:**
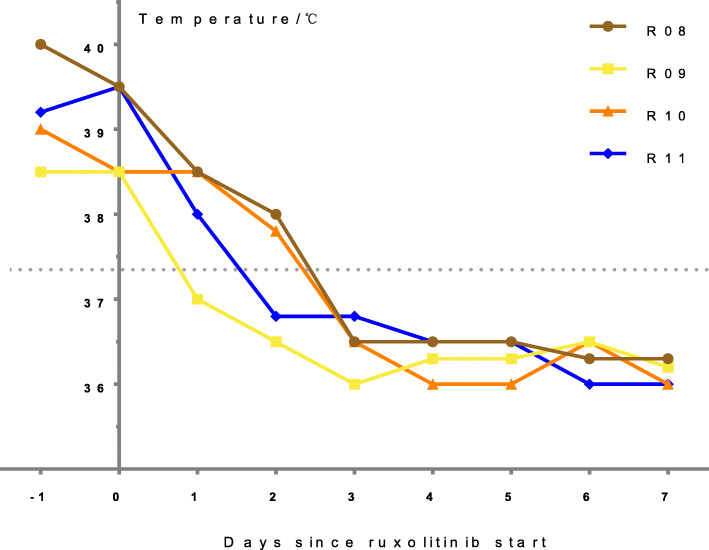
Body temperature of patients (P8–11). Body temperature returned to normal after 3 days of ruxolitinib treatment

The body temperatures of R1-R3 did not decrease after MP pulse treatment and intravenous immunoglobulin (IVIG) therapy (1 g/kg for 2 days), but returned to normal levels following administration of ruxolitinib. The temperature of R3 rose again after 3 days of ruxolitinib therapy but stabilized once the dose was increased from 2.5 mg bid (twice daily) to 3.75 mg bid (Fig. 1). Although the body temperatures of R4–R7 decreased to normal levels after conventional treatment, but they had severe organ involvement, including obvious liver damage and neuropsychiatric symptoms. They were all ameliorated by ruxolitinib treatment. R8–R11 were treated with ruxolitinib immediately after diagnosis. All children had normal body temperatures at 3 days after treatment (Fig. 2).

### Changes in body temperature between the two groups

**Fig. 3:**
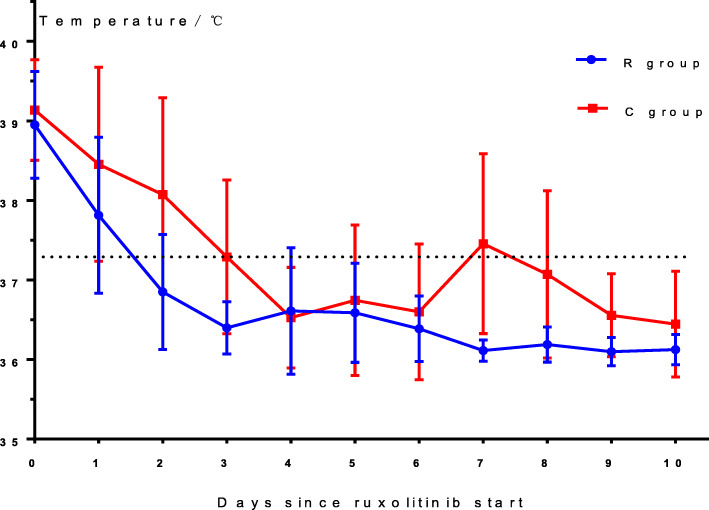
Mean body temperature of the two groups. Body temperature returned to normal after 3 days of ruxolitinib treatment. The mean body temperature of group R patients was lower than that of group C patients

There were no significant differences in body temperature between the two groups before treatment (*P* = 0.24). In group R, the children’s body temperatures decreased to normal levels after 2 days of treatment and remained stable. In group C, the body temperatures of eight patients decreased to normal levels after 3 days of treatment, whereas the body temperatures of the other three cases returned to normal levels after 5–6 days of treatment. However, fever recurred in eight patients in group C at 2–7 days after the body temperature had normalized and had to be controlled with immunosuppressants. Figure 3 shows the rapid temperature decrease in group R patients compared to group C patients: the temperature of group R patients was significantly lower than that of group C patients on days 2–3 and 7–9 after treatment (*P*d2 = 0.022, *P*d3 = 0.014, *P*d7 = 0.003, *P*d8 = 0.020, *P*d9 = 0.031).

### Changes in laboratory values (Table 2 and Fig. 4)

Following treatment with ruxolitinib, the white blood cell (WBC) count and fibrinogen levels gradually increased, whereas ferritin and IL-2R levels gradually decreased. The differences in WBC, fibrinogen, ferritin, and IL-2R levels of the two groups were significant compared to prior treatment. One week and 1 month after treatment, the WBC levels in group R patients showed significantly rapid improvement compared to those in group C patients (*P*1w = 0.037, *P*1m = 0.002). There was no significant difference in the ferritin levels of the two groups of patients (*P*1w = 0.398, *P*1m = 0.064). Although there were no significant differences in the fibrinogen and IL-2R levels of the two groups after 1 week (*P*1w distribution was 0.74, 0.062), these levels showed significantly rapid improvement in R group patients compared to those in group C patients after 1 month (*P*1m distribution was 0.035, 0.041). In group R, five cases of EBV infection, one case of HBV infection, one case of cytomegalovirus infection, and one case of influenza virus infection were treated with a combination of ruxolitinib and antiviral drugs (ganciclovir and entecavir), after which these patients tested negative for antiviral IgM antibodies and their viral DNA copy numbers had decreased. In group C, there were 2 cases with Epstein-Barr virus infection and 2 cases with parainfluenza virus infection. After traditional therapy combined with antiviral drugs (ganciclovir and oseltamivir), the clinical symptoms improved, the antiviral IgM antibodies turned negative, and the DNA copy number decreased.

**Table 2:** Changes in laboratory indices before and after treatment

Laboratory index	Group	Before treatment	1 week after treatment	1 month after treatment	F	P
WBC (×10^9^/L)	R	2.55 ± 2.21	5.45 ± 3.32	5.74 ± 2.52	8.896	0.002
	C	4.29 ± 3.37	8.77 ± 4.69	9.62 ± 5.17		
Fibrinogen (g/L)	R	0.9 ± 0.35	1.65 ± 0.66	2.17 ± 0.89	86.247	< 0.001
	C	1.36 ± 0.49	2.62 ± 0.21	3.94 ± 1.45		
Ferritin (μg/L)	R	17,090 ± 17,586	828.1 ± 646.4	178.9 ± 166.1	22.493	< 0.001
	C	12,579 ± 18,748	4765.6 ± 4562.2	597.6 ± 783.3		
IL-2R(U/mL)	R	9381.3 ± 12,865.4	2540 ± 1381.5	494.2 ± 195.8	5.146	0.036
	C	1784.5 ± 516.4	760 ± 155.6	390 ± 84.8		

*WBC* white blood cell; *IL-2R* interleukin-2 receptor; *Group R* ruxolitinib-treated group; *Group C* control group treated with conventional therapy. *P*-values describe comparisons within each group before and after treatment

**Fig. 4:**
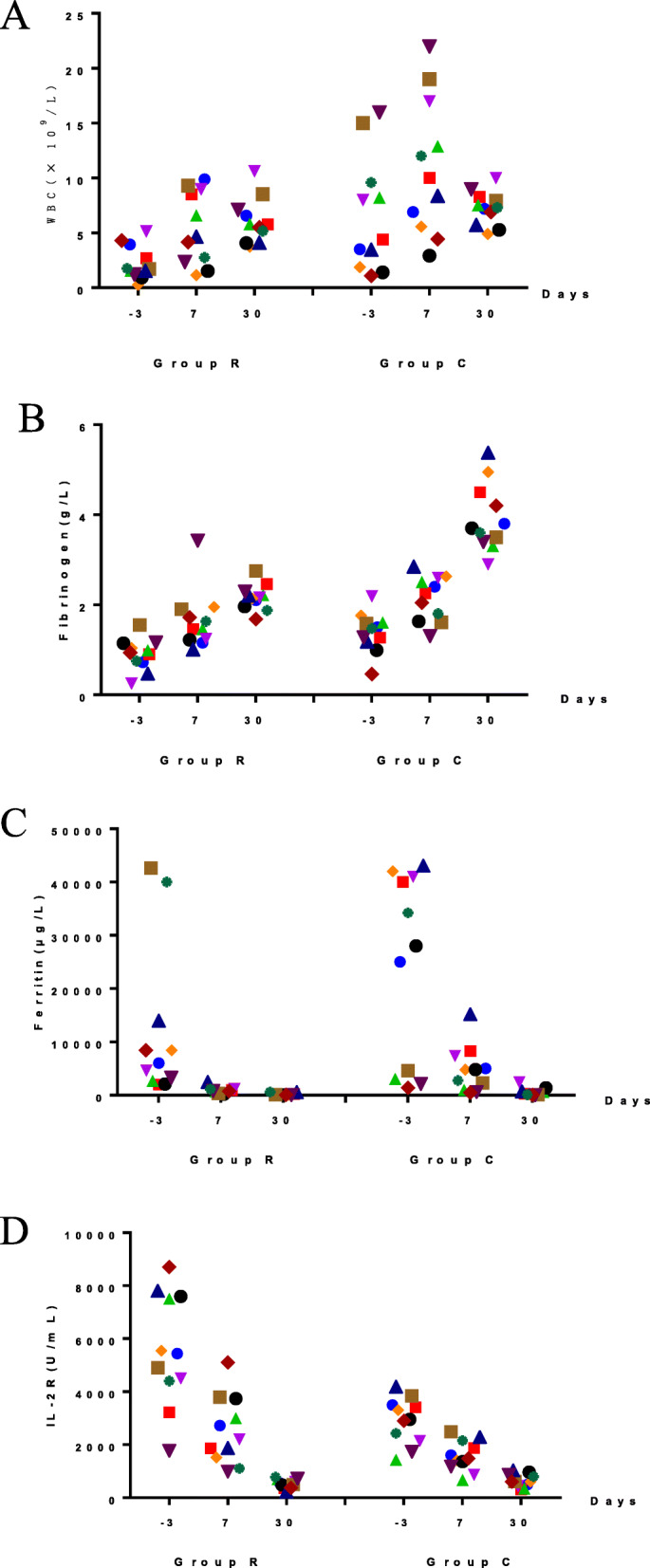
Comparison of changes in clinical indicators before and after treatment in the two groups. **a**-**d**) Levels of white blood cells, fibrinogen, ferritin and IL-2R, which are all inflammatory markers. Normal range values of WBC, fibrinogen, ferritin, and IL-2R are ≤10 × 10^9^/L, ≤ 4 g/L, ≤ 500 μg/L and ≤ 6400 pg/mL, respectively. The numbers on the x-axis of each graph represent the number of days before and after treatment

### Glucocorticoid dosage (Fig. 5)

The glucocorticoids used in both groups were given at equivalent doses of methylprednisolone (MP), and were always given at a lower dose in group R than in group C. After discharge, the MP doses were adjusted according to the laboratory indicators monitored. One and 2 months after discharge, the oral doses of MP in both groups were significantly lower than those at discharge (F = 60.536, *P* < 0.001). Compared with group C, the average dose of MP in children in group R was significantly reduced (F = 29.756, *P* < 0.001).

**Fig. 5:**
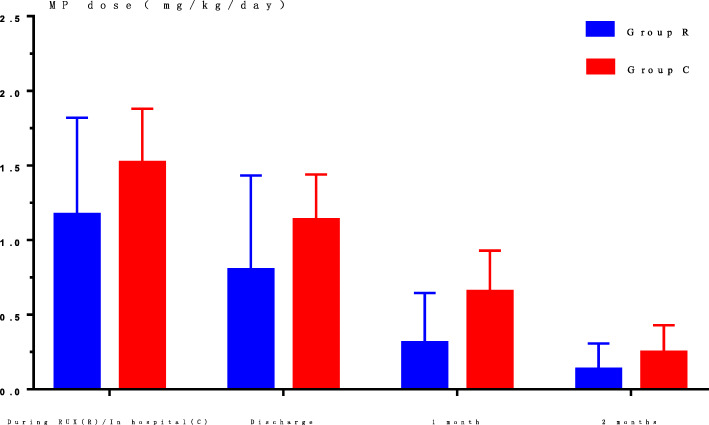
Comparison of MP doses between the two groups. Compared with group C, the average dose of MP in group R was significantly reduced (*P* < 0.01). Abbreviations: MP, methylprednisolone; RUX, ruxolitinib; Group R, ruxolitinib-treated group; Group C, control group treated with conventional therapy

### Safety evaluation of ruxolitinib

All children tolerated ruxolitinib well, with no dose-reductions or interruptions to ruxolitinib observed. Patients reported no headaches, dizziness, gastrointestinal adverse events such as nausea, vomiting, or gastritis, oral ulcers, sweating or constipation. Renal function was normal. One patient had mildly elevated liver enzymes after treatment, but this was resolved quickly by oral administration of bicyclol tablets.

### Follow-up

The patients were followed up for 2–2.5 years (average of 2.4 years). In group R, the glucocorticoid dose was first reduced, and the use of ruxolitinib was discontinued after 3 months of administration. Follow-up was continued for 21–27 months. One child presented with recurrent fever, accompanied by joint swelling and pain, and was diagnosed with juvenile idiopathic arthritis; the child improved after treatment with tocilizumab. In group C, three children with juvenile idiopathic arthritis experienced recurrent symptoms after glucocorticoid reduction, presenting as fever with joint swelling and pain; they also improved after treatment with tocilizumab.

## Discussion

In this study, we found that administration of ruxolitinib to children with HLH was effective for controlling their body temperature, improving inflammatory indices (ferritin, IL-2R), and ameliorating symptoms of CNS involvement. Combining glucocorticoids and antiviral agents resulted in the resolution of viral infection and reduction in the dose of glucocorticoids.

Hermans et al. found that administration of JAK inhibitors significantly inhibited the degranulation of mast cells and reduced the production of cytokines in an in vitro study of lymphocytes [39]. Ruxolitinib also reversibly improved the killing and degranulation of NK cells and ameliorated organ damage in HLH animal models [40, 41]. In 2016, Das et al. used lymphocytic choriomeningitis virus to infect perforin-deficient mice and construct a model of secondary HLH. A large dose of ruxolitinib (90 mg/kg) not only improved the disease symptoms and decreased cytokine levels in HLH model mice, but also increased the survival rates in mice [42, 43]. A small dose of ruxolitinib (1 mg/kg) also significantly improved long-term survival and clinical symptoms and promoted liver tissue regeneration [2]. Subsequently, a case of an 11-year-old child with refractory HLH who was treated with a combination of dexamethasone, etoposide, ruxolitinib (2.5 mg), and alemtuzumab was reported. Inflammatory factor levels rapidly decreased, organ function was restored, and no HLH relapse was observed even after etoposide treatment was discontinued [33]. Ruxolitinib was used as first-line treatment with dexamethasone in a 71-year-old patient with HLH. Administration of ruxolitinib (10 mg/dose, twice daily) was started on the 8th day of hospitalization; the patient’s condition and laboratory values improved on the 15th day of hospitalization [34]. A 38-year-old patient was treated with dexamethasone, immunoglobulin**,** etoposide, and rituximab; after administration of ruxolitinib (20 mg/dose, twice daily), there was an improvement in the patient’s phagocytic indicators such as serum ferritin, lactate dehydrogenase, and fibrinogen levels, but the patient eventually died of intracranial bleeding and multiple organ failure [35].

In this study, refractory cases of HLH were treated with ruxolitinib, resulting in the regulation of body temperature and inflammatory factors and improvement of CNS involvement. In addition, administration of ruxolitinib reduced the glucocorticoid dose, which is important for the normal growth and development of children.

Five cases (R1–R5) in group R had recurrent fever and showed no improvement in clinical features or inflammatory indicators such as IL-2R, ferritin, and C-reactive protein levels despite hormonal, immunosuppressive, and immunoglobulin therapies; hence, ruxolitinib was added to their treatment regimen. Their body temperature subsequently dropped rapidly and inflammatory indicators improved, demonstrating that ruxolitinib controlled the body temperature and inhibited the inflammatory response.

Two cases (R6, R7) with CNS involvement showed no improvement in neuropsychiatric symptoms after termination of the HLH-04 regimen, but gradually recovered with administration of ruxolitinib, suggesting a positive effect with CNS involvement. However, other studies have shown unsatisfactory therapeutic effects of ruxolitinib in patients with HLH combined with CNS involvement, attributing the lack of efficacy to its large molecular weight which prevents its penetration across the blood-brain barrier to act on the CNS [44]. This contradictory evidence warrants further study.

Four cases (R8–R11) were treated with ruxolitinib directly without using the HLH-04 regimen. One patient was diagnosed with HLH and HBV (published in the Journal of Pediatrics). As immunosuppressants and glucocorticoids may aggravate HBV infection, the child was treated with ruxolitinib combined with antiviral therapy using entecavir. All disease indicators improved significantly. During follow-up after more than 1 year, the body temperature of the child remained stable, and HBV-DNA was undetectable because of the treatment with antiviral drugs. This is the first study to use ruxolitinib alone to treat HLH. The other three children also had normal body temperature after treatment with ruxolitinib, and all indicators showed improvement.

Compared with group C, the dosage of glucocorticoids in R group children was apparently reduced throughout the treatment, suggesting that ruxolitinib can reduce the dosage of glucocorticoids or even replace glucocorticoid therapy, thereby reducing or avoiding glucocorticoid-related adverse reactions.

In children with co-infections, including EBV and HBV, there was no exacerbation of existing infections during ruxolitinib administration combined with antiviral therapy.

Possible side effects in children include oral ulcer, sweating, nausea, gastritis decreased appetite, thrombocytopenia, anemia, elevated liver enzymes and bilirubin, and hypertriglyceridemia. During the 2–2.5 years of follow-up, no children in group R had obvious adverse reactions. One patient developed fever again following discontinuation of ruxolitinib and had joint swelling and pain. That patient was diagnosed with juvenile idiopathic arthritis, and the patient’s condition improved after treatment with tocilizumab.

## Conclusion

To date, there have been a few reports of individual cases being treated with ruxolitinib. In this study, we present 11 cases of children treated with ruxolitinib, demonstrating that ruxolitinib might be effective for treating HLH and is convenient to administer. It may be used as a first-line treatment for HLH with glucocorticoid reduction. Treatment with ruxolitinib also improved the symptoms of inflammatory factors and CNS involvement in refractory HLH. A limitation of this study was retrospective, and the sample size was small. In future, prospective studies with large sample cohort should be used to confirm the safety, optimal dosage, treatment duration, withdrawal criteria, long-term effects, and efficacy of ruxolitinib in HLH caused by different etiologies. The findings of this study indicate that ruxolitinib permits lower cumulative glucocorticoid dosing, partially replaces glucocorticoids and becomes a potential first-line drug for the treatment of HLH, which is important for the normal growth and development of children.

## Data Availability

The datasets used and/or analyzed during the current study are available from the corresponding author on reasonable request.

## Abbreviations

HLH: Hemophagocytic lymphohistiocytosis
JAK: Janus Kinase
TH cell: T helper cell
NK cell: Natural killer cell
IL-2R: Interleukin-2 receptor
T-SPOT: T-cell enzyme immuno-spotting
EBV: Epstein-Barr virus
HBV: Hepatitis B virus
WBC: White blood cell
ESR: Erythrocyte sedimentation rate
CRP: C-Reactive protein
MP: Methylprednisolone
IVIG: Intravenous immunoglobulin
Group R: Ruxolitinib-treated group
Group C: Control group treated with conventional therapy
